# Molecular Genetics of Intracranial Meningiomas with Emphasis on Canonical Wnt Signalling

**DOI:** 10.3390/cancers8070067

**Published:** 2016-07-15

**Authors:** Nives Pećina-Šlaus, Anja Kafka, Mirna Lechpammer

**Affiliations:** 1 Laboratory of Neuro-Oncology, Croatian Institute for Brain Research, School of Medicine, University of Zagreb, Salata 12, Zagreb 10000, Croatia; anja.kafka@mef.hr; 2 Department of Biology, School of Medicine, University of Zagreb, Salata 3, Zagreb 10000, Croatia; 3 Department of Pathology & Laboratory Medicine, University of California, Davis, Medical Center 4400 V Street, Sacramento, CA 95817, USA; mlechpammer@ucdavis.edu

**Keywords:** meningioma, Wnt signalling, meningioma genetics, E-cadherin, APC, beta-catenin, AXIN1, p53

## Abstract

Research over the last decade recognized the importance of novel molecular pathways in pathogenesis of intracranial meningiomas. In this review, we focus on human brain tumours meningiomas and the involvement of Wnt signalling pathway genes and proteins in this common brain tumour, describing their known functional effects. Meningiomas originate from the meningeal layers of the brain and the spinal cord. Most meningiomas have benign clinical behaviour and are classified as grade I by World Health Organization (WHO). However, up to 20% histologically classified as atypical (grade II) or anaplastic (grade III) are associated with higher recurrent rate and have overall less favourable clinical outcome. Recently, there is emerging evidence that multiple signalling pathways including Wnt pathway contribute to the formation and growth of meningiomas. In the review we present the synopsis on meningioma histopathology and genetics and discuss our research regarding Wnt in meningioma. Epithelial-to-mesenchymal transition, a process in which Wnt signalling plays an important role, is shortly discussed.

## 1. Introduction

In the recent years, genome- and exome-wide sequencing approaches revealed a number of gene mutations and pathways associated with meningioma initiation and progression. Despite recent advances in understanding molecular, genomic and epigenetic profile of meningiomas, one of the most common intracranial tumours, further understanding of the factors that drive meningioma formation and progression is needed for successful treatment and subsequent clinical outcome. Most meningiomas are benign, indolent, slowly growing tumours, effectively treated with gross total surgical resection. However, up to 20% of the cases will have aggressive behaviour with high propensity for recurrence, which leads to an increased morbidity and mortality [1]. Even meningiomas that lack histological features of malignancy can recur. The origin of meningioma lies in progenitor cells that give rise to arachnoidal cap cells positioned outside of the thin arachnoid layer that covers the brain and spinal cord (Latin *arachnoidea encephali*; *arachnoidea spinalis*). The layer was termed arachnoid since its thin trabeculae form delicate web resembling a spider web (Latin *aranea*) [2]. Arachnoidal cap cells are a high metabolically active subgroup of arachnoid cells involved in the reabsorption of cerebrospinal fluid. In order to fulfil their functions, arachnoid cells form a variety of cell junctions [3]. To maintain strong adhesion these cells contain numerous desmosomes. Nevertheless, gap, tight, intermediate junctions and hemidesmosomes are present on these cells and play a role in adhesion. On the other hand, they also need flexibility which is provided by adherens junction molecule E-cadherin. Since arachnoid lacks vascularization intercellular circulation is very important. Metabolites and ions communicate through gap-junctions. The reason why meningioma cells are thought to be derived from arachnoid cells is that both cells share many ultrastructural and functional features. For instance desmosomes, tight junctions, pinocytic vesicles and cistern-like extracellular spaces are morphological features found in both cells [2].

The biological territories that need to be investigated in meningiomas are diverse. Currently, there is a lack of understanding of adhesion, migration, cell-to-cell communication, proliferation, differentiation, cell survival, apoptosis and tissue homeostasis in pathogenesis of meningioma. New research shows that multiple signalling pathways including Wnt pathway are involved in formation and growth of meningiomas. Today, a lot of expectation is placed on high throughput techniques and analyses which can provide us with knowledge about specific disease on a large scale basis. In this article we focus on recent molecular aspects of meningioma genetics and pathology and discuss several signalling pathways involved, including Wnt pathway. We will also give an overview of our research findings on the role of Wnt signalling in meningioma and comment on its potential role in epithelial-to-mesenchymal transition (EMT).

## 2. Epidemiology and Histopathological Classification

**Figure 1:**
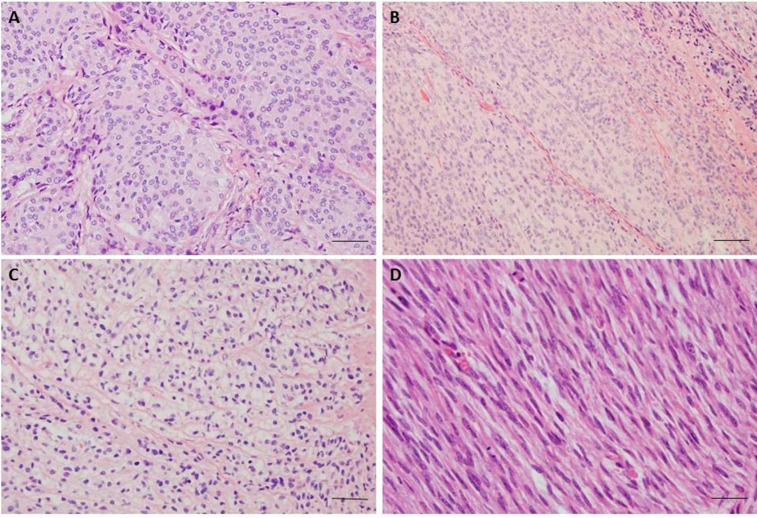
(**A**) Meningothelial meningioma, Grade 1; H & E staining, 200× magnification, showing typical whorl formations; (**B**) Atypical Meningioma, Grade 2; H & E staining, 200× magnification, with increased cellularity, sheet-like growth, high nuclear/cytoplasmic ratio and prominent nucleoli; (**C**) Clear cell meningioma, Grade 2; H & E staining, 200× magnification, meningothelial cell neoplasm with predominant clear, glycogen-rich cytoplasm; (**D**) Anaplastic Meningioma, Grade 3; H & E staining, 200× magnification, showing sarcoma like morphology and frequent mitoses. The scale bar 50 μm.

Sixteen different variants or subtypes of meningiomas are classified into three grades according to 2007 WHO classification [4,5,6]. The majority of meningiomas (80%) correspond to grade I and thus are considered to be benign, slowly growing tumours [4,5]. Grade I meningiomas exhibit a wide range of histological subtypes, including meningothelial, fibrous (fibroblastic), transitional (mixed), psammomatous, angiomatous, microcystic, secretory, lyphoplasmacyte-rich and metaplastic subtypes (Figure 1). Yet, these classifications are not always specific in respect to prediction of patient outcome, recurrence or response to treatment. Although most meningiomas histologically classified as “benign” have indolent biological behavior when adequately resected, there is substantial morbidity associated with recurrence. Meningiomas associated with less favourable clinical outcome, significantly higher rates of recurrence, morbidity and mortality correspond to grade II (atypical) and grade III (anaplastic). The incidence of atypical meningioma is 10%–15% and the incidence of anaplastic is fortunately low with 2%–5% of cases.

Atypical meningiomas are defined by histopathological criteria and two histological variants (clear cell and chordoid). Criteria include presence of at least 4 mitosis per 10 high power fields (HPF) or presence of at least three of the following histological features: sheet-like growth, spontaneous necrosis, high nuclear-to-cytoplasmic ratio, prominent nucleoli and increased cellularity. Anaplastic (grade III) meningiomas have excessive mitotic index defined as 20 or more mitoses per 10 high power fields (≥20/10 HPF) and the presence of frank anaplasia, including malignant cytology resembling carcinoma, melanoma or sarcoma. The brain invasion is a criterion for atypical meningioma [7]. Papillary and rhabdoid variant have been classified as variants of grade III. Aggressive meningiomas are usually highly vascularized and express high levels of vascular endothelial growth factor (VEGF). Current treatment options for recurring higher-grade tumours are inadequate [4,6,8,9].

The incidence of meningiomas is different in adults and children. Meningiomas account for approximately 30% of adult CNS tumors, whereas in children and adolescents incidence is 4.6% [10]. The mean age at diagnosis is 63 years and the incidence increases with age. The dramatic increase happens after age 65 and continues to be high even among the population aged 85 years and older. The overall annual incidence is approximately 7 per 100,000 individuals [4,11]. The incidence is increasing in the past 10 years and this trend is especially pronounced in females [11]. Meningiomas are incidentally found in 2.3% of performed autopsies. Meningiomas show great predominance in women in most investigated ethnic groups, with a female to male ratio of approximately 2:1 for intracranial and 10:1 for primary spinal meningiomas. The observed female predominance guides to the hypothesis that sex hormones could also be a risk factor for meningioma development. Meningioma frequently express estrogen and progesterone receptors from which half seem to be functional [12]. Since meningiomas exhibit steroid receptors on the cell surface, hormonal influences may explain the sexually dimorphic characteristics of this disease [13,14]. In favour of this hypothesis are the findings that meningiomas show increased growth during pregnancy or during hormone replacement therapy [15]. Also, ter Wengel et al. [16] found three patients who developed a meningioma in male-to-female transgenders. However, definite role of sex hormones as risk factors in the development and biology of meningiomas still needs to be established [16]. Besides estrogen and progesterone receptors, meningiomas express androgen receptors too, as well as nonsteroidal hormone receptors including receptors for somatostatin and dopamine [14].

Cytogenetic studies gave further insight into chromosomal alterations in meningiomas. A study by Tabernero et al. [17] analysed the characteristics of meningiomas in male and female patients by interphase fluorescence in situ hybridization (iFISH) and found the existence of different patterns of chromosome abnormalities and gene-expression profiles associated with patient gender. Male patients had a significantly higher percentage of del(1p36), while loss of an X chromosome was significantly associated to meningiomas from female patients. The group also showed a higher frequency of chromosome losses, other than monosomy 22, in meningiomas arising in male patients, while female patients displayed a higher frequency of chromosome gains or monosomy 22 alone. Eight genes displayed a significantly different expression pattern in male versus female patients and they were all localized to the sex chromosomes: two in chromosome X (DDX3X, XIST); and six in chromosome Y (RPS4Y1, DDX3Y, JARID1D, EIF1AY, USP9Y, and CYorf15B) [17].

The firmly established risk factor associated with development of meningiomas is primarily ionizing radiation. This environmental risk factor is usually associated to radiotherapy for primary intracranial tumours in childhood [18,19,20]. It has been shown that radiation-induced meningiomas are highly proliferative and more commonly high grade. Surprisingly, radiation-induced meningiomas rarely display NF2 alterations, both allelic losses and mutations in comparison to sporadic cases which may indicate that NF2 plays a less important role in the pathogenesis of radiation-induced meningiomas [18]. Besides, ionizing radiation, head trauma, hormone-replacement therapy and advanced age are also the established risk factors [21]. The use of mobile phones does not seem to be associated with the increased risk of meningioma development although there are controversial reports [9,22].

Survival rates of meningioma patients differ according to assigned grade [10,20], the 5-year overall survival is 92% for grade I, 78% for grade II and 47% (37.7% according to Champeauy et al. [23]) for grade III meningioma. Benign meningiomas have recurrence rates of ~7%–25%; atypical 29%–52% and anaplastic 50%–94% [5]. In the study by Perry et al. [24], tumour recurrence rates were 7%–20% of benign (Grade I), 29%–40% of atypical (Grade II), 50%–78% of anaplastic (Grade III) meningiomas. Age at diagnosis has a significant effect on relative survival: 10 years survival for ages 24–44 is 85.2% and for patients older than 75 it is 29.1% [11]. The risk of meningioma recurrence depends on multiple clinical and biological factors, histological grade, extent of surgical resection, tumour size, location, patient age and gender, increased mitotic activity, as well as genetic characteristics of the tumour [25]. Espinosa et al. [26] analysed the cytogenetic relationship between primary and subsequent recurrent meningiomas developed in the same individual and found that in most cases similar tumour cell clones identified in the initial lesion were also detected in the subsequent recurrent tumour samples. The authors concluded that the development of recurrent meningiomas after gross total tumour resection is usually due to regrowth of the primary tumour and rarely to the emergence of an unrelated meningioma. A prognostic signature for meningioma prognosis was reported by Chen et al. [27] who analysed genome wide expression profiles of 119 meningioma samples from two previously published DNA microarray studies [17,28] using the Cox proportional hazards regression models and found 37 genes to be specifically related to meningioma overall survival.

The current treatment and management options for meningioma patients include observation and surgical resection. It is important to individualize treatments for each patient, but the majority of patients are treated with surgery. Radiotherapy is reserved for some special circumstances or as adjuvant therapy. Chemotherapy is rarely utilized, since effective chemotherapeutic agents for meningioma are still in the investigation phase [2].

## 3. Genetics and Signalling Pathways

### 3.1. Chromosome Aberrations

Besides being histologically heterogeneous, meningiomas are also showing great cytogenetical heterogeneity. Chromosome gains and losses have been found to occur frequently. The most common alteration observed in meningiomas is monosomy of chromosome 22, observed in 40%–70% grade I meningiomas (WHO, 2007). Other most common cytogenetic alterations in meningioma, besides abnormalities in the 22q locus, are the deletion of the short arm of chromosome 1 (specific regions 1p33-34 and 1p36), loss of chromosomes 6, 10, 14, 18 and 19 [6,29,30] and gains of chromosomes 1q, 9q, 12q, 15q, 17q and 20q of which many are associated with tumour grade [21,31]. Besides 22q, losses of 6q, 10 and 14q have been proposed as particularly important events in meningioma progression and recurrence [4,6,32].

Sayagues et al. [33] applied multicolor iFISH analysis in a series of meningioma patients, using specific probes for DNA sequences of 11 chromosomes in combination with flow cytometry in order to explore the intratumoral cytogenetic heterogeneity of meningiomas. The group found that benign tumours displayed different intratumoral clonal evolution pathways from atypical/anaplastic meningiomas. Another study [1] proposed 3 major cytogenetic profiles: diploid, isolated monosomy 22 and complex iFISH karyotypes based on the cytogenetic characterization and unique protein expression profiles.

### 3.2. Gene Mutations and Gene Expression Analysis

Meningiomas are a principal feature of neurofibromatosis type 2 (NF2), a rare autosomal dominant disorder caused by germline mutation in the NF2 gene on 22q12.2. The first insight on genetic susceptibility for meningioma came from studies of such rare genetic syndromes. The main genetic event in this disorder is the biallelic inactivation of NF2 tumour suppressor gene and consequential loss of expression of its protein product. Up to 75% of patients with neurofibromatosis type 2 develop meningiomas during their lifetime [9]. The NF2 gene was identified in 1993 [34,35,36], and the protein it codes was named Merlin, also known as moesin-, ezrin-, radixin-like protein, showing a great sequence homology to members of the band 4.1 families of cytoskeleton-associated proteins [37,38,39]. Loss of merlin is a consistent finding in all NF2 associated meningiomas and meningiomas can be considered as tumours evolved due to the loss of merlin, together with schwannomas, hamartomas and ependymomas. Nevertheless, sporadic cases [22,37] exhibit similar genetic alterations in the 60% of cases. Mutations of NF2 in sporadic meningiomas show relatively high frequency and usually lead to a truncated protein [40]. Tabernero et al. found mutations which involved five different exons in 30% of sporadic meningiomas they investigated.

It has been shown that different WHO meningioma grades display similar frequency of NF2 inactivation which suggests that NF2 changes are early event in the etiology of this tumour. On the other hand different histologies display different frequencies of NF2 mutations. The NF2 mutations are frequently found in fibrous, transitional and psammomatous but also in atypical and anaplastic meningiomas, whereas meningothelial, secretory and microcystic subtypes rarely harbour NF2 mutations. It has been shown that meningothelial meningiomas tend to express lower levels of merlin loss than fibrous and other forms of meningiomas [41,42].

The definite list of gene alterations in meningiomas with unaffected NF2 gene remains unknown and is under intensive investigations. The high through-put data in cancer is providing us with information often referred to as genomic landscapes of cancer [28,43,44,45]. This term illustrates the multitude of specific genetic events in these complex diseases [46]. So what is the genomic landscape of meningioma? On the basis of recent whole genome-sequencing approaches [17,22,28,47,48,49,50], novel candidates in benign meningiomas have been identified which include: TRAF7 (the receptor-associated factor 7), KLF4 (Kruppel-like factor 4), AKT1 (v-akt murine thymoma viral oncogene homolog 1) and SMO (Smoothened). TRAF7 located on chromosome 16p13 is encoding a proapoptotic E3 ubiquitin ligase and the mutations of this gene occur in 24% of meningiomas.

The KLF4 gene located on chromosome 9q, is encoding 3 C_2_H_2_ zinc finger motifs and has been shown to be mutated in 10% of meningiomas. A persistent mutation in codon 409 of KLF4 gene’s exon 4 (K409Q) changing the wildtype lysine by a glutamine amino acid has been found to appear in parallel with TRAF7 mutations. It has been shown that KLF4 mutations were exclusive for secretory meningiomas so finding combined TRAF7/KLF4 mutation can serve in the diagnosis of secretory meningioma subtype [9].

SMO is encoding a negative regulator of the Hedgehog pathway and the mutations were found in 3%–5% of grade I meningiomas. AKT1 is encoding a key effector of PI3K signalling and has been found to be mutated in 10%–15% of meningiomas. AKT1 gene harbours an activating mutation (p.Glu17Lys) named AKT1E17K. A fraction of meningiomas (~13%), most frequently targeted with this mutation, were WHO grade I meningothelial and transitional meningiomas. Tumours grade II rarely harboured AKT1E17K mutation while it was absent in grades III [9]. 

A strong up-regulation of secreted frizzled-related protein 1 (SFRP1) expression was suggested in all meningiomas with AKT1E17K mutation. Therefore the use of SFRP1 immunohistochemistry may be a reliable marker for the detection of AKT1E17K mutations [51]. Abedalthagafi et al. [52] found oncogenic PI3K mutations in meningioma and demonstrated that they are as common as AKT1 and SMO mutations. Of note is that the mutations found in the above-mentioned genes are mutually exclusive of NF2 mutations.

Another gene thought to be involved early in meningioma pathogenesis and also being a member of the 4.1 family, is DAL1 with its gene product protein 4.1B [4]. Martinez-Glez et al. [53] performed a mutational study of DAL1 and found mutations in its exons 13 and 19, intron 18 and a polymorphism in exon 14. In approximately 60% of investigated meningiomas its reduced protein expression was found regardless of histological grade [9]. Lack of DAL1 protein was only slightly, and not significantly, more frequent in anaplastic meningiomas than in benign and atypical meningiomas, suggesting that it represents an early event in meningioma tumorigenesis. Combined loss of DAL1 and merlin was detected in 58% of investigated cases, suggesting that they belong to different signalling pathways [18].

In addition other candidate genes include SMARCB1 (INI1) involved in chromatin remodelling. The found germline missense mutation in SMARCB1’s exon 2 predisposes individuals to the development of multiple meningiomas and schwannomas [20,22]. Since loss of chromosome 22 region is a common event in meningioma, the region represents interesting genetic territory for search of additional candidate genes. Putative genes on chromosome 22q include LARGE (Like-Glycosyltransferase), BAM22 (AP1B1, Adaptor-Related Protein Complex 1, Beta 1 Subunit, also known as ADTB1 or beta-adaptin) and MN1 (Meningioma (Disrupted In Balanced Translocation) 1). LARGE encodes a member of the *N*-acetylglucosaminyltransferase gene family involved in the synthesis of glycoprotein and glycosphingolipid sugar chains. Also localized in the meningioma critical chromosomal region is BAM22. The gene codes for the subunit of clathrin-associated adaptor protein complex 1 [54], a member of the adaptin protein family. The molecule plays a role in protein sorting in the trans-Golgi network. BAM22 is part of the complexes that mediate both the recruitment of clathrin to membranes and the recognition of sorting signals within the cytosolic tails of transmembrane receptors. MN1 gene located on 22q12.1 [55,56] was found to be disrupted in its first exon by balanced translocation (4; 22) in a meningioma patient. Resent research [44,56] indicates that MN1 (a putative meningioma tumour suppressor) was found to be differently expressed in malignant and benign meningiomas. Chang et al. [44] assessed gene expression levels and copy number variants using microarray platform and showed that MN1 was significantly repressed in all the malignant samples analysed in their study. Zhang et al. [56] from exome sequencing data, identified two novel potential driver mutations in MN1 which nominated MN1 as a candidate gene for malignant transformation of meningiomas.

Other genes alterations associated with meningioma include the well-known TP53 gene. Although mutations of the TP53 gene have been reported to be rare in meningiomas, low frequency of point mutations is constantly found and reported [57,58,59]. In addition, relatively high incidences of somatic mutations and enhanced expression in meningiomas have been reported for sis, myc, ras, fos, mos, TP73, BCL-2 and STAT3 oncogenes [56].

Several genes have been associated with malignant progression in meningioma. Tumour suppressor genes CDKN2A (encoding p16^INK4a^), ARF (encoding p14^ARF^), and CDKN2B (encoding p15^INK4b^) residing on chromosome 9p21 are all associated with the anaplastic grade [30,38]. Homozygous deletions or mutations of the above mentioned genes are found in most anaplastic meningiomas [4,9].

Another candidate engaged in meningioma progression is the TIMP3 (the tissue inhibitor of metalloproteinase 3) gene on 22q12, because it has been shown that anaplastic meningiomas showed much higher hypermethylation of its promoter than atypical and benign cases [6]. The maternally expressed gene 3 (MEG3) located in the 14q32 region has been implicated in meningioma progression, too. Allelic losses, promoter hypermethylation and reduced expression of MEG3 gene have been associated to aggressive meningioma phenotype. It has been reported that MEG3 has anti-proliferative and anti-tumour activity in meningiomas. The gene encodes a non-coding RNA, whose expression was reduced in aggressive meningiomas [6].

Lusis et al. [60] using DNA microarray techniques identified a new meningioma associated candidate gene NDRG2 (N-myc downstream regulated gene 2) on chromosome 14q11.2. This tumour suppressor is a Myc-repressed gene and is supposed to participate in cell growth, differentiation and p53-mediated apoptosis [25]. NDRG2 was found to be commonly inactivated in meningioma progression. It is down-regulated in anaplastic meningiomas and atypical meningiomas with aggressive clinical behaviour. The reduced expression of NDRG2 was associated with promoter hypermethylation [6]. Skiriute et al. [25] observed statistically significant differences in NDRG2 gene expression level between primary and recurrent meningioma groups and between benign (WHO grade I) and atypical (WHO grade II) meningiomas measured at the mRNA level. Interestingly, NDRG2 contributes to the regulation of the Wnt signalling pathway. It down-regulates CTNNB1-mediated transcriptional activation of target genes, such as CCND1 and may thereby act as a tumour suppressor [61,62].

Genetic and expression alterations found in meningioma are systematized in Table 1.

### 3.3. Microsatellite Instability

The investigations into the mechanisms of the maintenance of genomic stability and integrity are also relevant in meningioma research field. The usual incidence rate of spontaneous somatic mutations is much lower to the rates of genetic changes observed in tumour cells and this increased frequency is the result of genomic instability that characterizes tumour cells. To simplify, the genome of tumour cells, besides the accumulation of somatic mutations is also affected by additional genomic instability. A type of genomic instability which reflects impaired cellular mismatch repair is microsatellite instability (MSI). MSI is associated with changes in the number of repetitive DNA sequences termed microsatellites. Simple repeated sequences are genetically unstable, as judged by their polymorphic nature in the human population [64]. Pećina-Šlaus et al. have found replication/repair machinery to be constantly targeted in the meningiomas they investigated in two different studies [65]. Microsatellite markers specific for two different Wnt genes that were used (CDH1, AXIN1), revealed a fraction of meningiomas with MSI. This is indicative of malfunctioning of replication/repair genes (hMLH1 or hMSH2, hPMS1, hPMS2), opening a potential area of interest in meningioma studies. D16S752 microsatellite tetranucleotide marker for E-cadherin gene (CDH1) revealed 11% of samples with MSI. All MSI samples were reamplified and repeatedly analysed on both Spreadex and polyacrylamide gels. The samples demonstrating MSI were confirmed by direct sequencing. One meningothelial, one transitional and one anaplastic case harboured MSI [65].

There are other studies that show that certain proportions of meningiomas demonstrate features of genomic instability. Pykett et al. [66] have reported that 25% of meningiomas exhibit MSI, Sobrido et al. [67] have reported on 6.3% of meningiomas with MSI at 2 or 3 loci, which is similar to the findings of Zhu et al. [68] of 2.4%. Bethke et al. [69] analysed single nucleotide polymorphisms of the DNA repair genes in association to meningioma predisposition and found that some DNA repair gene variants are connected to higher risk of meningioma development. The similar results on DNA repair variants affecting the risk of meningioma are reported by Rajaraman et al. [70]. A study by Chen et al. [71] investigated the roles of the methylation of hMLH1 and MSI in meningiomas and found 4.66% of cases to exhibit MSI. Hypermethylation of a promoter of hMLH1 was found in 18% of investigated meningiomas and associated to meningioma progression. In their paper Yang et al. [72] showed that a tumour suppressor gene—CHEK2, involved in DNA repair and genome stability, contributes to the genomic instability in meningiomas. Alternative splicing and frequent codeletion of CHEK2 with NF2 in meningiomas harbouring chromosome 22q deletions impaired DNA repair in their study and increased chromosomal instability, thus promoting meningioma progression.

### 3.4. Epigenetic Studies in Meningioma

New evidence suggests that altered epigenetic regulation which include: altered DNA methylation, microRNA expression, histone and chromatin modifications, plays an important role in the pathogenesis of meningiomas [21,73]. Of note, aberrant promoter hypermethylation of a variety of genes has been identified as a frequent event in atypical and anaplastic meningiomas, suggesting that epigenetic changes are substantially involved in meningioma progression [6]. The detailed description of these findings is beyond the scope of this article.

### 3.5. Signalling Pathways

Understanding the genetic basis and molecular etiology of meningioma is essential for clinical phenotype determination as well as patient outcome. The involvement of multiple pathways also suggests that therapy could be targeted against specific signalling level [74].

Molecular pathways driving meningioma progression still need elucidation. Our knowledge on specific genetic drivers of malignant transformation is also incomplete [75]. Novel findings [41,74,76,77] suggest that activation of multiple growth factor receptors and their signalling pathways are responsible for the growth of meningiomas.

One of the first gene expression profiling studies of meningiomas was performed by Watson and co-workers [78] who identified gene transcripts differentially expressed between normal leptomeningeal cells and meningiomas of different grades. Gene expression study by Tabernero et al. [17] showed a relationship of expression profiles to the cytogenetic subgroups of meningiomas and patient outcome. Domingues et al. [1] investigated the different protein expression profiles by immunophenotyping of individual meningioma cells and also found association with tumour cytogenetics.

Comparative tissue proteomic profiling of meningioma shed light on molecular basis of the tumorigenesis using another approach. A study by Sharma et al. [77] investigated alterations in tissue proteome in different grades of human meningiomas. Combining the results obtained from two mass spectrometric platforms, the authors identified 2367 proteins that exhibited differential expression in meningiomas. Functional analysis of the identified differentially expressed proteins confirmed the modulation of signal transduction pathways, including integrin signalling, Wnt signalling, Ras signalling, FGF signalling, EGF growth signalling, apoptosis signalling and ubiquitin proteasome signalling.

Merlin acts as a tumour suppressor and is capable of modulating a wide range of signalling pathways [79,80,81,82]. It interacts with cell-surface proteins, proteins involved in cytoskeletal dynamics and proteins involved in regulating ion transport. Merlin-lacking cells are also known to contain defective adherens junctions [39]. It has been shown that merlin inhibits signalling and the activation of downstream pathways, including the Ras/Raf/MEK, PI3K/AKT/mTOR, Rac/PAK/JNK and Wnt/β-catenin pathways [82].

Already from the listing of genes involved in meningioma in the previous paragraphs, we can assume which signalling pathways to suspect for meningioma development. Primarily involved is the notorious Ras/Raf/MEK signalling pathway. It has been shown that receptor tyrosine kinases such as epidermal growth factor receptor (EGFR) and platelet-derived growth factor receptor (PDGFR) are widely expressed in meningioma tumour cells. Equally important is the Pi3K/Akt/mTOR signalling pathway [9,47,74]. Both pathways lie downstream of receptor tyrosine kinases. Hilton et al. [74] have demonstrated the expression of phosphorylated Jnk and Mek, in addition to Erk, pS6RP and Akt, in the majority of meningiomas of all grades. Besides EGFR and PDGFR, the kinases known to be involved are Erb-B2 Receptor Tyrosine Kinase 2 (ERBB2), insulin-like growth factor 1 receptor (IGF1R) and vascular endothelial growth factor receptors (VEGFRs) [82]. Alterations in the RB retinoblastoma protein and p53 pathways are presumed because of the dysregulation of p16INK4a, p15INK4b, and p14ARF. The involvement of TGFbeta/SMAD as well as Hedgehog pathways is also noted as important [38]. Integrin mediated signalling via Rac/PAK/JNK [83,84] has proved to be particularly interesting too. Studies linking the NF2 tumour suppressor as a modulator of growth factor and extracellular matrix signals that trigger Rac1-dependent cytoskeleton-associated processes indicate its important role in the processes of cell adhesion and migration.

Insulin-like growth factor (IGF) signalling cascade [85] has been shown to be involved too, since both IGF-II and IGFBP2 are expressed in meningiomas, with increased concentrations of IGFII associated with invasiveness and malignant progression [4]. Gene expression transcript profiling study [86] revealed the deregulation of Notch pathway [73].

## 4. Wnt Signalling

Signalling pathways build complex molecular network within the cell and their accurate functioning maintains cellular homeostasis. Signal transduction pathways which regulate cell survival, proliferation and migration are also fundamental in tumorigenesis. Alongside other well-known signalling pathways is Wnt signalling pathway, primarily studied in development which regulates key intercellular signalling events during embryogenesis [87,88] and plays an important role in the development of central nervous system. Components of Wnt signalling regulate multiple aspects of brain development in vertebrate embryos. Wnt ligands have been identified as key regulators of regional identity in the early developing of the forebrain [89]. They modulate axon pathfinding, dendritic development and synaptic assembly [90]. Yu and Malenka [91] identified beta-catenin, the Wnt pathway’s main effector signalling molecule, as a critical mediator of dendritic morphogenesis. Specifically, overexpression of a stabilized beta-catenin in transgenic neural precursors causes massive expansion of the cerebral cortex, while loss-of-function mutations in individual Wnts cause deletions or malformations of distinct brain regions [92]. A study by Lang et al. [93] showed that adenomatous polyposis coli (APC), yet another key component of Wnt signalling, enhances proliferation of oligodendroglial progenitor cells (OPCs). It is known that lymphoid-enhancer factor 1 (LEF1) and Tcf4 (Transcription Factor 4; T cell factor 4), pathway’s transcription factors, are required for proneural and neuronal gene expression, for neuronal differentiation in the posterior hypothalamus [94] and for oligodendrocyte differentiation [95]. Huang et al. [96] reported that Wnt pathway co-receptors, Lrp 5 and 6, are required for the development of the cerebellum.

The pathway has two distinct branches, the canonical (or beta-catenin) and non-canonical (or planar cell polarity (PCP) and Wnt-Ca^2+^ pathways). Among two Wnt signalling cascades the canonical is the longest known and the best studied one [97].

**Figure 2:**
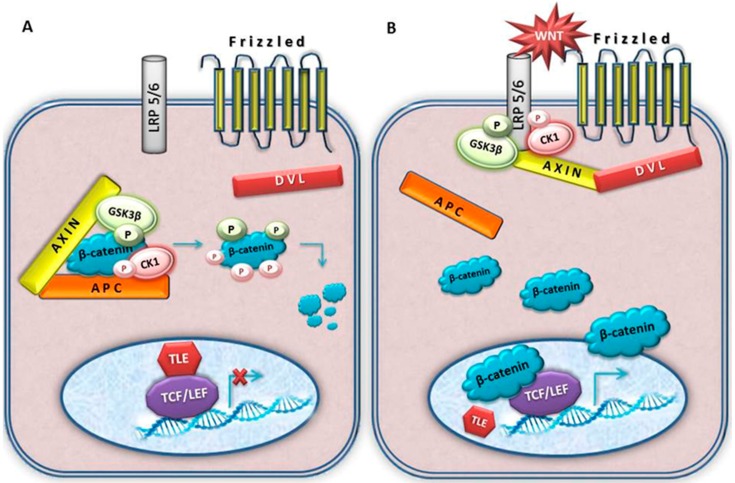
A schematic illustration of the canonical Wnt signal transduction cascade. Panel (**A**), in the absence of Wnt ligand, a destruction complex consisting of AXIN, APC, GSK3-β and CK1 resides in the cytosol. β-catenin is phosphorylated by CK1 and GSK3-β and targeted for degradation by the proteosomal machinery; Panel (**B**), with Wnt stimulation, some components of protein complex dislocate from the cytosol to the plasma membrane. The destruction complex falls apart and β-catenin is stabilized. Dvl is also recruited to the membrane and binds to Fz and Axin which is bound to phosphorylated LRP5/6. Stablized β-catenin is translocated to the nucleus where it associates to LEF/TCF transcription factors, displacing co-repressor TLE and recruiting additional co-activators to Wnt target genes. The activated Wnt pathway is associated to meningioma.

The pathway has two modes, active and inactive (Figure 2). When inactive, levels of beta-catenin are downregulated and kept low. This is achieved by beta-catenin destruction complex where beta-catenin is being phosphorylated by glycogen synthase kinase 3 beta (GSK3β) and casein kinase 1 (CK1). The phosphorylated beta-catenin is targeted for quick ubiquitinilation and degradation in the proteosome. Beta-catenin cellular levels are regulated by capturing it in the destruction complex where AXIN serves as a backbone. Besides AXIN, the complex is also composed of APC, GSK3β and CK1 [88,98,99]. Once bound to this protein complex, beta-catenin is sequentially phosphorylated on four relevant amino acids: serines 45, 37 and 33; and threonine at 41, ultimately resulting in the targeting of beta-catenin for degradation [100,101].

The pathway is active in response to Wnt ligands, highly conserved secreted signalling molecules. In this mode beta-catenin cannot be degraded and it accumulates in the cytoplasm. Upon cytoplasmic stabilization it enters the cell nucleus and since it is unable to bind to the DNA lacking necessary domains it finds a partner among members of the DNA binding protein family LEF/TCF. Bound to such transcription cofactors it impacts the expression of target genes including cyclin D1, c-myc, fra-1 and c-jun.

The main characteristic of Wnt signalling activation is the rise of beta-catenin cytosolic levels. This is achieved with the help of Dishevelled proteins that bind AXIN, pulling it out of the destruction complex and taking it to the cell membrane. There, DVL will form large molecular supercomplexes consisting of Wnt-Fz-LRP5/6-DVL-AXIN. When AXIN is no longer part of beta-catenin destruction complex, the complex is destroyed and beta-catenin can no longer be degraded.

Mutations that enable constitutive activation of the Wnt pathway can be responsible for malignant transformation of a cell. For example, mutations in the beta-catenin gene have been reported in a variety of human tumours. Most mutations found in different types of tumours are located in exon 3, the so called mutational hot spot. Exon 3 codes for the part of beta-catenin where the serine/threonine residues, important for phosphorylation, are situated. Therefore, if this region is mutated, beta-catenin can no longer be degraded; it stabilizes firstly in the cytoplasm, then is transferred to the nucleus. It is interesting to note that in the absence of beta-catenin TCF binds to the repressor of the pathway, Groucho, in vertebrates transducin-like enhancer of split (TLE); and acts as a co-repressor of Wnt target genes [87]. As already mentioned earlier, the malfunctioning of the pathway has repeatedly been implicated in a number of human tumours.

## 5. Key Wnt Signalling Molecules Involved in Meningioma

A number of novel studies indicate that one of the important signalling pathways targeted in meningioma is the Wnt pathway [6,28,73,77]. Watson and co-workers [78] identified gene transcripts differentially expressed between nonmalignant leptomeningeal cells and meningiomas and found that Frizzled (Wnt receptor) had 3.7 times fold increased levels in meningioma. The survey by Wrobel et al. [102] observed that anaplastic meningiomas could be distinguished from benign by differential expression of a distinct set of genes and suggested that their behaviour could be governed by different genetic profiles. The authors found four genes linked to the Wnt signalling: beta-catenin (CTNNB1), the regulatory subunit of cyclin-dependent kinase 5 (CDK5R1), ectodermal-neural cortex 1 (ENC1) and cyclin D1 (CCND1), with the increased expression in meningiomas examined by microarray. CCND1 and ENC1 were also upregulated in anaplastic meningiomas as compared to benign cases in their study.

A study by Domingues et al. [1] analysed the different protein expression profiles in meningioma and proposed 3 major cytogenetic profiles: diploid, isolated monosomy 22 and complex iFISH karyotypes. The intranuclear role of CTNNB1 as transcription cofactor was connected to IGF2 and part of complex karyotype tumours in their study.

It has been shown that Secreted Frizzled-Related Protein 1 (SFRP1), a member of a family of soluble proteins known for their ability to inhibit the Wnt signalling pathway plays a role in meningioma recurrence [73,103]. The validation of the microarray expression data for SFRP1 confirmed significantly lower mRNA levels in recurrences than in original meningiomas (*p* < 0.05) [103]. However, SFRPs can also be upregulated in other tumours. It has previously been shown that astrocytomas [104] have decreased SFRP3 expression in the nucleus, finding that positively correlates with increasing astrocytoma grade; whereas in the cytoplasm the increase in SFRP3 protein expression was associated with higher grade astrocytomas. It seems that SFRP3 can act both as an antagonist and agonist of the Wnt signalling depending on the cellular context. Therefore further studies are needed to elucidate the role of SFPR1 in meningioma.

Ludwig et al. [105] identified 13 miRNAs deregulated between different subtypes of benign meningiomas and 52 miRNAs deregulated in anaplastic meningioma when compared with benign meningiomas. Known and putative target genes of deregulated miRNAs found in their study include genes involved in Wnt signalling and in epithelial-to-mesenchymal transition for benign meningiomas. Papers by Chang et al. [44] and He et al. [73] report results of gene expression levels and copy number variants in benign, atypical and malignant meningiomas. Many Wnt signalling components have been found as targets in this study, for instance, TCF3, SFRP3, SFRP1, CDH1, FZD7 (Frizzled Class Receptor 7).

Sharma et al. [77] in their tissue proteome study of meningioma found Wnt signalling cascade as one of the significantly modulated pathways. Frizzleds, Casein Kinase 1 Alpha 1 (CSNK1A1), also known as CK1 and SFRPs were all upregulated, while serine/threonine protein phosphatase B (PP2A) was downregulated.

Pecina-Slaus et al. investigated the involvement of Wnt signalling pathway in meningioma by analysing its key signalling molecules, APC, beta-catenin, E-cadherin and AXIN1. They showed [106] significant association between APC genetic changes and lack of wild type protein expression, or presence of mutant APC proteins in meningiomas indicating involvement of this tumour suppressor gene. Thirty-three meningiomas were analysed regarding genetic changes of this tumour suppressor gene. Two genetic markers, Rsa I in APC’s exon 11 and Msp I in its exon 15 were used to test genetic changes using the polymerase chain reaction/loss of heterozygosity (LOH) and RFLP method. Gross deletions of the APC gene were found in 47% of investigated meningiomas. The observed genetic changes of the APC gene were dispersed among different types of benign meningioma, indicating that APC is not likely to be the first event in the advancement of this tumour. Meningiomas that were harboring LOHs were also accompanied with the absence of APC protein expression or presence of mutant APC proteins (Chi square = 13.81, df = 2, *p* < 0.001).

APC changes also influenced beta-catenin expression and nuclear localization. Beta-catenin was upregulated and transferred to the nucleus in 71.2% of meningiomas and its nuclear localization correlated to gross deletions of APC gene (Chi square = 21,96, df = 2, *p* < 0.0001). This high frequency of nuclear transfer is indicative of beta-catenin’s importance in the biology of meningioma.

Together with APC in the β-catenin destruction complex is a scaffold protein AXIN1, functioning as a tumour suppressor in cancer. AXIN1, 16p13.3, 96 kDa, is an inhibitor of Wnt signalling. It down-regulates beta-catenin by facilitating its phosphorylation by GSK3-beta. It binds directly to APC, beta-catenin, GSK3-beta and Dishevelled [107,108]. There is emerging evidence suggesting that AXIN plays critical roles in the regulation of synaptic functions, formation of synaptic protein complexes and anchoring postsynaptic proteins in the central nervous system. LOH of AXIN1 gene was found in 21.1% of meningiomas. The majority of investigated samples showed moderate or strong (78.2%) levels of expression for AXIN1. Nevertheless, seven out of 32 samples (21.9%) demonstrated negative or very weak AXIN1 expression levels with exclusive cytoplasmic localization when compared to the levels of AXIN1 in healthy brain tissues. Strong statistical correlations were observed between cytoplasmic localization of AXIN1 and its weak expression; and also between the simultaneous cytoplasmic and nuclear localizations; and moderate and strong expression levels (*p* < 0.000) [109].

E-cadherin (gene CDH1 at 16q22.1, encodes a 120-kDa glycoprotein) is considered an indirect modulator of Wnt signalling. Bound to beta-catenin, it is localized on the surfaces of cells in regions of cell-cell contacts known as adherens junctions, while its intracellular domain interacts with the actin cytoskeleton. The downregulation, or loss of E-cadherin expression is considered responsible for dysfunction in cell-cell adhesion. We assume that the disruption of bound beta-catenin can rise cytoplasmic levels of this molecule and thus indirectly modulate the activation of Wnt signalling.

The results of analysis on E-cadherin in meningiomas showed downregulation or loss of its protein expression in 73% of the total meningioma samples investigated [65]. Downregulation observed in meningioma subtypes was in 50% of meningothelial, 80% of fibrous, 80% of transitional, 90% of angiomatous, 80% of atypical and in 80% of anaplastic. Gross deletions of the CDH1 gene were also detected in 32% of investigated meningiomas. Altogether nine samples with LOH of the CDH1 gene out of 28 heterozygous patients were observed with the gross deletions distributed as follows: 2 in 11 informative meningothelial meningiomas; 4 in 6 informative fibrous; 3 in 4 informative angiomatous. Next, significant association between the genetic changes of CDH1 and the nuclear localization of beta-catenin protein was found (Chi square = 5.25, df = 1. *p* < 0.022).

The results on E-cadherin in meningioma by other authors show similar patterns of expression. Schwechheimer et al. [110] found that E-cadherin’s expression was absent from the majority of malignant meningiomas they examined and Utsuki et al. [111] also reported on negative E-cadherin immunostaining of their meningioma sample. Brunner et al. [112] believe that it is unlikely that loss of NF2 expression is associated with loss of the proper localization of beta-catenin and E-cadherin in meningiomas. Saydam et al. [113] evidenced that miR-200a has a role in meningioma growth via E-cadherin and Wnt/beta-catenin signalling pathway. Downregulated miR-200a in meningiomas promoted tumour growth by reducing E-cadherin and activating the Wnt pathway. A direct correlation between the downregulation of MiR-200a and the upregulation of beta-catenin was demonstrated in this study.

Recently a concomitant expression of beta-catenin and p53 was investigated [63]. The involvement of two signalling pathways in meningioma was questioned and analysed by their main effector molecules; beta-catenin for Wnt signalling and p53 for p53 signalling. An interconnection between the Wnt and p53 pathways in cancer has previously been suggested [114]. Our analyses showed that 47.5% of the total sample demonstrated loss of expression of p53 protein while beta-catenin was upregulated in 71.2% of meningiomas. The levels of the two proteins were significantly strong, negatively correlated in the analysed meningiomas (*p* = 0.002). This indicates that meningiomas with lost p53 upregulate beta-catenin and activate Wnt signalling. Similarly, Sadot et al. [115] demonstrated the dose-dependent reciprocal relationship between beta-catenin and p53 expression in the H1299 cells. The summary of the molecular findings of the differentially expressed genes of Wnt signalling pathway found in meningiomas is shown in the following Table 2.

## 6. Epithelial-to-Mesenchymal Transduction

Wnt signalling is strongly involved in epithelial-to-mesenchymal transition. This process is essential for embryogenesis where EMT enables controlled and precise movement of cells in gastrulation and neural crest formation. Cells that are till then closely held together acquire fibroblast resemblance and start to move individually. When cells gain such migratory mesenchymal phenotype they become plastic and able to perform conversions between epithelial-to-mesenchymal and mesenchymal-to-epithelial transitions (MET). In embryonic development cells undergo many rounds of EMT and MET.

Similar to embryonic development EMT takes place during tumour progression and metastasis. Here loss of tissue integrity leads to local invasion where previously noninvasive tumour cells acquire motility, leave the tissue parenchyma, enter the systemic circulations and ultimately disseminate to distant organs. The reverse process of MET enables the migratory cells to acquire epithelial phenotype once they reach their destination in order to form metastasis.

Although extremely uncommon it has been reported that meningiomas can metastasize. Metastatic dissemination of malignant meningiomas can be intra-cranial or to distant organs. Similar to other malignant neoplasms, malignant meningiomas can also infiltrate neighbouring tissues and form metastatic deposits [4]. It has been reported that extracranial meningiomas (0.1% of cases) can metastasize to lungs, liver, pleura, bone and kidney locations. The lung is the most frequently involved site. Brain invasion and intra-cranial dissemination can also occur and could be explained by molecular mechanisms of EMT. Although metastatic spread of meningioma is more likely to occur in WHO grades II and III, grade I lesions can also metastasize [4].

The molecular mechanisms driving meningioma invasion are still not well understood. It is hypothesised that the events of EMT may play role in it. Meningiomas display both mesenchymal and epithelial characteristics [116]. The meninges at the skull base are derived from the mesoderm, and the telencephalic meninges are derived from the neural crest [9]. One of the known functions of arachnoidal cap cells is the production of collagen and fibroblast-like ECM proteins. Kida et al. [117] have shown the presence of tight junctions in arachnoid cells lining the arachnoid granulations. The expression of E-cadherin in the arachnoid membrane, arachnoid granulations and meningioma has been confirmed by IHC [3]. The most prominent feature of EMT is the loss of expression of the cell-cell adhesion molecule E-cadherin and it has been observed in many carcinomas [118]. It is known that E-cadherin can be inactivated through many different mechanisms including deletions and mutation, transcriptional repression as well as its promoter hypermethylation. The mechanism that keeps adherens junctons strength is the amount of cadherin molecules present at the cell membrane.

The phenomenon that happens in EMT during normal development, the so called cadherin switch, has been known to happen in meningiomas too. In this phenomenon, E-cadherin is replaced by N-cadherin and in tumours it is regarded as a sign of invasive behaviour. Therefore E-cadherin is considered as an invasion suppressor gene. Another important molecular event involved in EMT is beta-catenin’s translocation to the nucleus [119,120,121]. The stabilization and nuclear accumulation of beta-catenin can induce EMT because it can enhance the expression of two transcriptional repressors SNAIL1 and SNAIL2 (also known as SLUG). It has been demonstrated that SNAIL1 and SNAIL2 can bind to E-cadherin promoter region and repress its expression, thus weakening adherens junctions and inducing EMT [122,123,124]. As discussed in the previous paragraphs beta-catenin was progressively upregulated and transferred to the nucleus in our study on meningiomas.

The migratory mesenchymal cells when having reached their target destinations have the genetic potential to revert to their original epithelial phenotype through a process known as MET. The incitement of the mechanisms of MET in invasive or metastasing cells is a tempting idea for the therapeutic approaches in cancer, including aggressive meningioma.

## 7. Conclusions and Future Perspectives

The identification of molecular markers of meningioma recurrence is essential for clinical phenotype determination as well as patient outcome.

The synthesis of knowledge on genetics, cellular signalling pathways, histopathological phenotypes and clinical parameters will lead to the classification of individual patient’s tumour according to signature alterations, with a final goal of subsequent development of novel and successful treatment options in the dawn of personalized and precision medicine. The observed pathways involved in meningioma etiology will provide opportunities to improve prognostic markers for meningioma and predict clinical behaviour, recurrence and response to therapy.

New research indicates that alongside other well-known signalling pathways also stands the Wnt pathway with important roles in meningioma formation and progression.

## Figures and Tables

**Table 1:** Genetic and expression alterations reported in meningioma.

Affected Genes and Their Locations	MA or ES **	Expressional Changes *	Meningioma Grade	Tumorigenesis	Citations
PI3K/3q26	MA	↑	Grade I	Early event	[52]
SMO/7q32.1	MA	↑	Grade I	Early event	[17,22,28,47,48,49,50]
KLF4/9q31	MA	↓↑	Grade I	Early event	[17,22,28,47,48,49,50]
AKT1/14q32.33	MA	↑	Grade I	Early event	[17,22,28,47,48,49,50]
TRAF7/16p13	MA	unknown	Grade I	Early event	[17,22,28,47,48,49,50]
DAL1/18p11.32	MA; ES	↓	Grade I, II, III	Early event/Progression	[4,6,9,18]
SMARCB1/22q11.23	MA	↓	Multiple meningioma	Early event	[20,22]
NF2/22q12.2	MA; ES	↓	Grade I, II, III	Early event	[9,22,34,35,36,37]
BAM22/22q12.2	MA	↓	Grade I, II, III	Early event	[6,38,54]
CDKN2A/9p21	MA	↓	Grade III	Progression	[4,6,9,30,38]
ARF/9p21	MA	↓	Grade III	Progression	[4,6,9,30,38]
CDKN2B/9p21	MA	↓	Grade III	Progression	[4,6,9,30,38]
NDRG2/14q11.2	MA; ES	↓	Grade II, III	Progression	[6,25,60]
MEG3/14q32	MA; ES	↓	Grade III	Progression	[6]
TP53/17p13.1	MA; ES	↓↑	Grade I, II, III	Progression	[57,58,59,63]
MN1/22q12.1	MA; ES	↑↓	Grade I, II, III	Progression	[38,44,55,56]
LARGE/22q12.3	MA	↓	Grade I, II, III	Progression	[4,38]
TIMP3/22q12	MA; ES	↓	Grade III	Progression	[6]

* ↓ = downregulated; ↑ = upregulated; ** MA = mutational anlysis; ES = expression studies.

**Table 2:** Differentially expressed genes of Wnt signalling pathway found in meningiomas.

Gene	Locus	Product	Function	Deregulation	Meningioma Effect	Citation
FZD2	17q21.1	Frizzled class receptor 2	receptor for Wnt signaling proteins	upregulation	tumorigenesis	[78]
FZD7	2q33	Frizzled class receptor 7	receptor for Wnt signaling proteins	upregulation		[44,73]
CSNK1A1	5q32	Casein kinase 1, alpha 1	transferring phosphorus-containing groups protein tyrosine kinase activity	upregulation	tumorigenesis	[77]
APC	5q22.2	Adenomatous polyposis coli	negative regulator of Wnt signaling tumor suppressor	loss of heterozygosity	tumorigenesis	[106]
AXIN1	16q13.3	Axin1	negative regulator of Wnt signaling tumor suppressor	gross deletions, downregulation, MSI	cell growth and tumor progression	[107,108]
CTNNB1	3p22.1	β-catenin	key downstream component of the canonical Wnt signaling transcription cofactor	upregulation	cell growth and tumor progression associated to complex karyotype meningiomas	[1,63,73,102,114,115]
PPP2CA	4q24	Serine/threonine protein phosphatase 2B	negative control of cell growth and division	downregulation	tumorigenesis	[77]
TCF3	19p13.3	Transcription factor 3 (T cell factor 3)	transcription factor	upregulation	tumorigenesis	[44,73]
CCND1	11q13.3	Cyclin D1	regulator (progression through) of cell cycle	upregulation	cell growth and tumor progression	[28,102]
ENC1	5q13.3	Ectodermal-neural cortex 1	role in the oxidative stress response a role in malignant transformation	upregulation	cell growth and tumor progression	[102]
FRZB (SFRP3)	2q32.1	Secreted frizzled-related protein 3	modulator of Wnt signaling	upregulation	tumorigenesis	[44]
SFRP1	8p11.21	Secreted frizzled-related protein 1	tumor suppressor	downregulation,	recurrence	[28,41,44,73,102,103]
				upregulation	when AKT1E17K mutation is present	
CDH1	16q22.1	E-cadherin	regulator of cell-cell adhesions	Downregulation loss of function, gross deletion, MSI	cell growth and tumor progression	[65,73,78,110,111,112,113]
NDRG2	14q11.2	N-myc downstream regulator 2	transcription factor tumor suppressor	Downregulation, promoter hyperventilation	cell growth and tumor progression	[6,25,61,62]
CDK5R1	17q11.2	Cyclin-dependent kinase 5, regulatory subunit 1	G1/S transition of mitotic cell cycle, development of the central nervous system	upregulation	cell growth and tumor progression	[73,102]
